# Individual Differences in Sensitivity to Everyday Sounds: Developmental, Environmental, and Personality Predictors of Auditory Processing

**DOI:** 10.7759/cureus.88141

**Published:** 2025-07-17

**Authors:** Akemi Hara, Yume Otsuki, Keita Shimizu, Michio Murakami, Yasuhiro Kotera, Akihiko Ozaki, Taichi Akutsu

**Affiliations:** 1 Research, Medical Governance Research Institute, Minato, JPN; 2 Early Childhood Studies, Okayama Prefectural University, Soja, JPN; 3 Center for Infectious Disease Education and Research, The University of Osaka, Suita, JPN; 4 Psychology, Faculty of Medicine and Health Sciences, University of Nottingham, Nottingham, GBR

**Keywords:** acoustic stimulation, auditory perception, child development, personality traits, social environment

## Abstract

Background

Sensitivity to everyday sounds shows considerable individual variation. This study aimed to identify distinct dimensions of auditory sensitivity and examine how psychosocial and developmental factors, such as early auditory environments and personality traits, shape individual differences in non-clinical adults. In addition, we explored sensitivity to low-salience sounds that are typically perceived as non-intrusive by most people but elicit discomfort in a minority of individuals.

Methods

A cross-sectional web-based survey was conducted between September and November 2024 with 380 Japanese adults (aged 18-56), using a self-developed questionnaire informed by prior research and pilot testing. Participants rated their affective reactions to 39 everyday sounds. Factor analysis extracted latent auditory sensitivity components. Multiple regression analyses examined the predictive roles of childhood auditory environment and personality traits measured via standardized psychological scales.

Results

Based on factor analysis of participants’ ratings across 39 everyday sounds, two underlying dimensions of auditory sensitivity were identified. These were labeled WLS1 and WLS2, reflecting different patterns of sensitivity: WLS1 represents sensitivity to subtle, non-salient sounds, while WLS2 reflects heightened reactivity to loud or sharp auditory stimuli. WLS1 was significantly predicted by childhood exposure to parental vocal discipline (β = 0.19, 95% confidence interval (CI) (0.07-0.31), p = 0.002), emotional reactivity (β = 0.23, 95% CI (0.09-0.36), p = 0.001), and interpersonal assertiveness (β = 0.24, 95% CI (0.10-0.37), p < 0.001). WLS2 was associated with female gender (β = 0.50, 95% CI (0.27-0.72), p < 0.001), insomnia from worry (β = 0.15, 95% CI (0.04-0.27), p = 0.006), fatigue (β = 0.30, 95% CI (0.17-0.43), p < 0.001), and impulsivity (β = 0.14, 95% CI (0.04-0.24), p = 0.008). A third factor, W1, reflecting sensitivity to ambient mechanical sounds that over 80% of participants rated as non-intrusive, was predicted by orderliness (β = 0.16, 95% CI (0.03-0.25), p = 0.012), emotional sensitivity (β = 0.21, 95% CI (0.08-0.34), p = 0.002), and a preference for uniqueness (β = 0.29, 95% CI (0.15-0.44), p < 0.001).

Conclusions

Auditory sensitivity is multifactorial and shaped by both developmental auditory experiences and personality dimensions. The results suggest that distinct psychological pathways underlie responsiveness to subtle versus intense auditory stimuli.

## Introduction

Auditory sensitivity refers to the degree to which individuals experience heightened or disproportionate perceptual or emotional responses to everyday environmental sounds. It is not uniformly distributed across individuals; rather, it reflects a spectrum of perceptual experiences shaped by both biological predispositions and lived environments [1-3]. While some sounds, such as alarms or construction noise, are widely perceived as intrusive, others, like the ticking of a clock or quiet conversations, are typically filtered out or tolerated by most [4,5]. However, a notable subset of individuals reports disproportionate discomfort even toward these low-salience sounds, suggesting that individual differences in auditory processing are not fully explained by stimulus characteristics alone [6-10].

This phenomenon has been studied globally under various labels, such as misophonia, noise sensitivity, and selective sound intolerance, with most research concentrated in North America and Europe [11,12]. These studies predominantly focus on clinically distressed populations, often linking heightened auditory sensitivity with emotional dysregulation, obsessive-compulsive traits, or increased physiological arousal [13-17]. Conceptually, Western research tends to frame auditory sensitivity as a neurophysiological vulnerability or a failure of emotional regulation [14,18]. More recently, some have adopted trait-based frameworks, such as sensory processing sensitivity, to account for broader patterns of multisensory sensitivity even in non-clinical individuals [14,19].

In contrast, Japanese research has primarily focused on children and adolescents, often within developmental or educational settings. Studies have linked auditory filtering difficulties to behavioral rigidity and social sensitivity in youth populations [20], and heightened startle responses to low-intensity sounds have been associated with social cognition traits [21]. Adult-oriented studies in Japan have largely concentrated on objective measures such as hearing thresholds or age-related decline, rather than subjective sensitivity or discomfort in everyday life [22]. This age-group skew highlights a research gap in understanding how auditory sensitivity manifests across the broader adult population.

These cross-cultural differences reflect distinct conceptual approaches: Western studies often pathologize auditory sensitivity by isolating conditions like misophonia [11-18], whereas Japanese research, though narrower in scope, frames it as part of a broader profile involving social vigilance or heightened awareness [20,21]. In both traditions, the emphasis remains on clinical or extreme cases, with limited attention to non-clinical variation in everyday life. As a result, we still lack a clear understanding of how everyday soundscapes may cause distress, not due to pathology, but through the interaction of personality, developmental history, and cultural conditioning [4,10,19]. This gap limits our ability to distinguish between clinically relevant sound sensitivity and normative individual variation and hinders the development of appropriate support strategies for non-clinical populations.

To address these gaps, this study treats auditory sensitivity as a multidimensional trait shaped by both developmental exposure and dispositional characteristics. Drawing on sensory processing sensitivity theory and developmental plasticity frameworks [19,23,24], we hypothesize that early auditory environments, from emotionally intense family interactions to structured musical training, play a crucial role in shaping how individuals later perceive, prioritize, and emotionally interpret environmental sounds. Furthermore, personality traits such as emotional reactivity, interpersonal vigilance, and need for order may mediate responses to low-salience but socially embedded sounds [25,26].

This study pursued two aims related to auditory sensitivity in adults aged 18 and older. First, we aimed to identify distinct dimensions of auditory sensitivity by examining individual differences in reactions to a broad range of everyday environmental sounds, while also exploring how early childhood auditory experiences may influence later auditory perception. Second, we investigated how these sensitivity profiles are associated with early childhood musical training and stable personality characteristics, with particular attention given to individuals who report discomfort toward low-salience sounds typically ignored by others.

## Materials and methods

Ethical considerations

This study falls outside the scope of the ethical guidelines for medical and health research involving human subjects issued by the Japanese government, as it does not involve clinical interventions or identifiable medical records. Currently, Japan does not have national ethical regulations specific to social and behavioral research involving adults. Accordingly, our study was conducted in accordance with the Ethical Principles for Sociological Research of the Japan Sociological Society, which does not require formal ethical review for anonymous, non-interventional survey research. In fact, when we made inquiries to the ethics committees of institutions where the authors are affiliated, such as Okayama Prefectural University and the Medical Governance Research Institute, we received similar responses. All participants were informed of the purpose of the study and voluntarily provided informed consent prior to participation.

Study design and setting

This study employed a cross-sectional observational design based on a self-administered online questionnaire. A convenience sampling framework was used to recruit participants and distribute the survey. The goal was to examine individual differences in auditory sensitivity among Japanese adults in relation to their early auditory environments and personality characteristics.

A total of 380 individuals aged 18 and older who provided informed consent participated in the study. This number closely approximates the required sample size of 384, calculated based on the Japanese population with a 95% confidence interval (CI) and a 5% margin of error, as suggested by Serdar et al. (2021) [27]. Participants were eligible if they were Japanese residents aged 18 years or older and provided informed consent. Individuals with known hearing impairments or ongoing psychiatric treatment were excluded from participation. The sample included individuals from various age groups, with the majority being between 20 and 29 years old. Specifically, 45 participants were aged 18 to 19, 314 were in their 20s, 13 were in their 30s, five were in their 40s, and three were in their 50s. The sample included 160 male participants, 217 female participants, and three individuals who did not report their gender. No additional demographic data were collected.

The study followed a structured timeline. In August 2024, prior to formal data collection, three university students were asked to review the questionnaire and provide verbal feedback to help refine the clarity and relevance of the items. This preliminary step was not part of the actual data collection but served as a pretesting process for improving the questionnaire design. Based on their suggestions, minor revisions were made to the wording of certain questions.

The finalized questionnaire was distributed between September 7 and November 1, 2024, using Google Forms and circulated via LINE (Line Corporation, Tokyo, Japan), a widely used messaging platform in Japan. One of the authors individually sent the link to approximately 250 acquaintances, primarily in their 20s, through personal LINE messages, including friends and former classmates from junior high and high school. Participants were further encouraged to share the link within their university group chats or with friends, leading to about 50 additional responses through indirect sharing. Around 100 more responses were obtained through a LINE open chat community created for mutual exchange among undergraduate thesis projects, where participants voluntarily answered each other’s questionnaires. Responses were collected from individuals across different regions of Japan. As participation was voluntary and the questionnaire was administered online, a response rate could not be calculated. The full set of items is provided in Appendix K.

Independent variables

Independent variables were selected based on prior research highlighting potential factors that influence auditory sensitivity. Specifically, early environmental exposure, early childhood musical training, and individual personality traits have been shown to shape sensory processing and sound perception during the developmental stage [27,28]. Based on these theoretical foundations, the questionnaire items were initially developed by the authors through repeated consultation and discussion among team members with relevant expertise. To further improve the clarity and relevance of the items, preliminary feedback was solicited from three university students, who were asked to review the draft questionnaire and comment on its comprehensibility and content. This feedback contributed to the refinement of item wording and structure. Through this process, the content validity of the survey was strengthened, ensuring that it appropriately captured demographic, experiential, and psychological dimensions considered important for understanding individual differences in auditory sensitivity.

Demographic variables

Demographic variables include gender (male/female) and age (18 to 56 years).

Q2: Early Childhood Musical Experiences (Seven Items)

"Q2" refers to the second item set in the self-developed questionnaire. The Q2 series evaluates early childhood musical exposure and engagement before elementary school. The total score ranges from 0 to 28, with higher scores indicating more frequent and diverse engagement in musical activities during early childhood. It includes seven items measuring the frequency and nature of early musical experiences. The first six items assess participation in music-related activities, recorded on a frequency scale from 0 (none) to 4 (daily). The seventh item measures formal music instruction, recoded into an ordinal variable based on the starting age: 4 (before age 3), 3 (ages 3-6), 2 (elementary school), 1 (middle school or later), and 0 (no instruction).

Q3: Preschool Environment (Eight Items)

The Q3 series assesses the auditory characteristics of the preschool environment and their potential impact on early auditory experiences. Each item was rated on a 5-point Likert scale ranging from 0 (strongly disagree) to 4 (strongly agree), with higher scores indicating greater exposure to auditory stimuli in preschool settings. This section includes items that examine children's exposure to auditory stimuli in preschool settings, such as the presence of loud speech from teachers and peers, engagement in group activities that involve sound, and reactions to various auditory elements within the learning environment.

Q4: Early Childhood Residential Environment (15 Items)

The Q4 series evaluates the early childhood home environment, focusing on auditory exposure within the household. Items were rated on a 5-point Likert scale from 0 (strongly disagree) to 4 (strongly agree), with higher scores reflecting greater auditory stimulation or vocal intensity in the home environment. Items in this section measure aspects such as parental speech volume, discipline-related vocal expressions, and the general auditory atmosphere of the home, including potential sources of stress or comfort associated with sound.

Q5: Personality Traits (50 Items)

The Q5 series explores individual personality traits that may be relevant to auditory perception and sensitivity. Each item was rated on a 3-point scale: 0 = no, 1 = not sure, and 2 = yes. Higher scores indicate stronger endorsement of the respective trait. This section includes items assessing tendencies related to emotional responsiveness, sensitivity to external stimuli, social behaviors, organizational habits, and general psychological dispositions. These traits may influence how individuals perceive and respond to different auditory environments.

Outcome variables

To examine individual differences in auditory sensitivity, participants were asked to evaluate 39 types of sounds that may trigger auditory discomfort. Of these, 35 items were adapted from Matsui and Sakuma (2020) [29], who developed a sound sensitivity scale in the context of auditory hypersensitivity among individuals with developmental disorders.

In addition, four original items were developed by the authors based on feedback from several university students, who were asked to reflect on and describe sounds they personally found disturbing or uncomfortable. Sounds that were not included in the original 35-item scale but were frequently mentioned during this feedback process, such as “whispering voices between others” and “shouting voices,” were added as original items. This resulted in a total of 39 sound items. All items were reviewed collaboratively by the research team to ensure content validity and to reflect contemporary everyday sound environments.

Q6: Auditory Environment and Sensitivity (39 Items)

The Q6 series measures individual differences in auditory sensitivity and experiences with environmental sounds. Each item was rated on a 4-point Likert scale ranging from 0 (not sensitive at all) to 3 (extremely sensitive), with higher scores indicating greater perceived sensitivity to the corresponding sound. This section includes items that examine respondents' sensitivity to various auditory stimuli encountered in daily life, such as mechanical noises, human speech, and environmental sounds. The responses provide insight into how individuals perceive and react to different types of auditory input in their surroundings. A complete list of Q6 items and their descriptions is available in Appendix K.

Statistical analyses

All statistical analyses were conducted using RStudio (Version 4.4.1; RStudio Team 2015, RStudio: Integrated Development for R. RStudio, Inc., Boston, MA, USA). A p-value of less than 0.05 was considered statistically significant.

Integrated multidimensional modeling of auditory perception: a factor-analytic and regression approach to environmental, musical, and personality determinants

Outcome Variable - Auditory Sensitivity Factor Scores

The primary outcome variables were weighted least squares (WLS) factor scores derived from factor analysis of the Q6 series. Participants rated their sensitivity to each of the 39 sounds using a 5-point Likert scale (0 = not sensitive at all, 4 = extremely sensitive), reflecting how noticeable or bothersome each sound was. These ratings enabled the identification of latent dimensions of auditory sensitivity. The resulting factor scores represent individual differences in auditory sensitivity and served as the dependent variables in subsequent regression analyses.

Independent Variable Domains

To examine potential predictors of individual differences in auditory sensitivity, three major independent variable domains were analyzed.

Early childhood musical experiences (Q2): A composite music experience score was computed by summing responses from seven items, with Cronbach’s alpha used to evaluate internal consistency.

Early auditory environmental exposure (Q3 and Q4): Q3 (preschool environment) and Q4 (early childhood residential environment) were combined and subjected to factor analysis to extract latent environmental factors representing early auditory environmental exposure.

Item Analysis and Factor Analysis Procedures

All items from Q3, Q4, and Q6 underwent rigorous item analysis, and only those with discrimination indices above 0.4 were retained [30,31]. Given the ordinal nature of the data, a polychoric correlation matrix was computed for both Q3+Q4 and Q6.

Assessment of data suitability: The suitability of the dataset for factor analysis was confirmed via the Kaiser-Meyer-Olkin (KMO) measure and Bartlett’s test of sphericity.

Determination of factor structure: The optimal number of factors was determined through parallel analysis, scree plot inspection, and theoretical interpretability, ensuring a statistically robust and conceptually meaningful structure [32].

Factor Extraction Methodology

Both factor analyses employed WLS estimation with oblimin rotation [33], ensuring a theoretically coherent structure. Items with factor loadings exceeding 0.30 were retained, while those with substantial cross-loadings (>0.30 on multiple factors) were removed to ensure factor distinctiveness [34]. Model fit was evaluated using the Comparative Fit Index (CFI), Tucker-Lewis Index (TLI), and Root Mean Square Error of Approximation (RMSEA).

Regression Analyses

Following factor extraction, factor scores were computed using regression-based estimates and merged into the dataset.

Core regression model: The extracted factor scores from Q6 served as the primary dependent variables, while demographic covariates (gender and age), factor scores from Q3 and Q4, and the composite Q2 music experience score were included as independent variables in subsequent regression analyses. The Variance Inflation Factor (VIF) was calculated to assess multicollinearity among predictors.

Extended analysis with personality traits: To explore the association between personality traits and auditory sensitivity, stepwise linear regression was conducted using the full set of Q5 personality traits as candidate predictors and W-series factor scores as dependent variables. The VIF was calculated for these models to assess multicollinearity. Model selection was based on the Bayesian Information Criterion (BIC), with both forward and backward selection employed.

Integrated multidimensional modeling of non-salient auditory stimuli: a factor-analytic and regression approach to individual sensitivity determinants

Outcome Variable - Sensitivity to Non-salient Auditory Stimuli

The secondary outcome focused on sensitivity to non-salient auditory stimuli. Specifically, these are Q6 items for which nearly 80% of respondents reported low sensitivity. These items represent sounds that are generally unnoticed or non-intrusive to most people but may still elicit responses in certain individuals. Factor analysis was applied to these items to derive the W series factor scores, which quantify individual differences in sensitivity to non-salient auditory stimuli.

Chi-Square Analysis

We identified the relevant Q6 items based on the high percentage (≧80%) of respondents reporting low sensitivity. These items were converted into categorical data, and chi-square tests were conducted to verify their statistical independence.

Item Analysis and Factor Analysis Procedures

Factor analysis followed the same procedure as described earlier, including polychoric correlation computation, data suitability tests, and factor extraction using WLS with oblimin rotation. The resulting W series factor scores were computed using regression-based estimates and merged with the dataset.

Regression Analyses

Stepwise linear regressions were conducted using the newly derived W-series factor scores as dependent variables and all Q5 personality traits as candidate predictors. To identify the most relevant traits, model selection was performed using the BIC with both forward and backward selection. This approach enabled the extraction of a parsimonious set of personality traits associated with sensitivity to non-salient auditory stimuli.

## Results

Participant characteristics

The final sample consisted of 380 participants aged 18-56. As shown in Table 1, the majority of participants were in their 20s (82.6%). Gender distribution included 160 males (42.1%), 217 females (57.1%), and three individuals (0.8%) who did not report their gender. No additional demographic variables were collected.

**Table 1:** Participant Demographics

Category	Subgroup	N (%)
Age group	18-19 years	45 (11.8)
	20-29 years	314 (82.6)
	30-39 years	13 (3.4)
	40-49 years	5 (1.3)
	50-59 years	3 (0.8)
Gender	Male	160 (42.1)
	Female	217 (57.1)
	Not reported	3 (0.8)
Total		380 (100.0)

Outcome Variable - Auditory Sensitivity Factor Scores (Q6)

All retained Q6 items demonstrated discrimination indices above 0.4, indicating strong item validity. The data were highly suitable for factor analysis (KMO = 0.89; Bartlett’s test: χ² (741) = 15716.25, p < 0.001). Parallel analysis, scree plot inspection, and theoretical interpretability supported a two-factor solution. A final set of 27 items was retained for analysis (Table 2 and Appendix B).

**Table 2:** Factor Loading of Auditory Sensitivity Items WLS1: sensitivity to subtle, non-salient sounds; WLS2: heightened reactivity to loud or sharp auditory stimuli

Item	WLS1	WLS2	Communality	Uniqueness	Complexity
Sounds of large groups talking or crowd noise	0.16	0.64	0.55	0.45	1.10
Sounds of campaign vehicles or advertising trucks	0.00	0.83	0.70	0.30	1.00
Construction site noise	-0.03	0.88	0.74	0.26	1.00
Motorcycle or car horns	-0.05	0.91	0.78	0.22	1.00
Sounds of a baby crying	0.22	0.46	0.37	0.63	1.40
Ambulance siren	0.24	0.54	0.48	0.52	1.40
Sound of a toilet air dryer	0.67	0.12	0.54	0.46	1.10
Sound from TV or radio	0.70	0.08	0.56	0.44	1.00
Sound of train brakes	0.20	0.61	0.54	0.46	1.20
Sound of a hairdryer	0.66	0.16	0.57	0.43	1.10
Background music in a store	0.86	-0.02	0.72	0.28	1.00
Indoor announcement	0.84	0.07	0.78	0.22	1.00
Sound of audio equipment	0.83	0.00	0.69	0.31	1.00
Sound of air conditioning or ventilation fan	0.82	0.00	0.68	0.32	1.00
Sound of a door opening or closing	0.70	0.13	0.60	0.40	1.10
Sound indicating a bus stopping or entering	0.73	0.18	0.69	0.31	1.10
Sound of a clock's second hand	0.75	0.00	0.56	0.44	1.00
Sound of a refrigerator	0.91	-0.06	0.78	0.22	1.00
Footsteps inside a house	0.73	0.04	0.57	0.43	1.00
Sound of flushing a toilet	0.89	0.00	0.80	0.20	1.00
Sound of sniffling	0.56	0.18	0.46	0.54	1.20
Sound of knocking on a door	0.80	0.03	0.67	0.33	1.00
Sound of water falling into a sink	0.86	-0.03	0.71	0.29	1.00
Sound of a cash register operation	0.97	-0.14	0.82	0.18	1.00
Sound of a traffic signal	0.93	-0.12	0.77	0.23	1.00
Sound of writing utensils during a test	0.58	0.17	0.46	0.54	1.20
Shouting voice	-0.05	0.78	0.57	0.43	1.00

Factor analysis was conducted on Q6 items to explore the underlying dimensions of auditory sensitivity. Items with discrimination indices above 0.4 (Appendix A) were retained for analysis. The data showed high suitability for factor analysis, with a KMO value of 0.89 and a significant Bartlett’s test of sphericity (χ² (741) = 15716.25, p < 0.001). Parallel analysis, inspection of the scree plot, and theoretical interpretability supported a two-factor solution for the Q6 items. Based on this, a final set of 27 items was retained for analysis (Table 2 and Appendix B).

The final model explained 64% of the total variance, with Factor 1 accounting for 46% and Factor 2 for 18%. The model demonstrated an acceptable fit, with a CFI of 0.844, a TLI of 0.816, and an RMSEA of 0.116 (90% CI: 0.111-0.122). Factor correlations indicated a moderate relationship between the two latent constructs (r = 0.51), supporting their theoretical distinction while suggesting some shared variance.

The two extracted factors represent different aspects of auditory sensitivity, specifically distinguishing between sensitivity to subtle, continuous environmental sounds and high-intensity, intrusive noises:

Factor 1 (WLS1) primarily reflects sensitivity to persistent background sounds, including ambient mechanical noises commonly present in daily life, such as air conditioning, ventilation, refrigerators, and indoor footsteps.

Factor 2 (WLS2) captures responsiveness to loud, intrusive external noises, including high-intensity auditory stimuli from public and urban settings, such as construction noise, motorcycle horns, ambulance sirens, and crowd noise.

After factor extraction, factor scores for WLS1 and WLS2 were computed for each participant and merged with the main dataset for subsequent regression analyses.

Independent variable domains

Early Childhood Musical Experiences (Q2)

First, the reliability analysis of Q2 (Early Childhood Musical Experiences) yielded a Cronbach’s alpha of 0.76, indicating acceptable internal consistency. Consequently, a composite Music Experience Score was computed for each participant by summing responses across the relevant items. This score serves as an index of how frequently and intensely participants engaged with musical activities in early childhood, which may influence later auditory processing.

Early Auditory Environmental Exposure (Q3 and Q4)

Nine items from Q3 (preschool environment) and Q4 (early childhood residential environment) with discrimination indices above 0.4 were retained for factor analysis (Appendix C). The data were suitable for factor analysis (KMO = 0.76; Bartlett’s test: χ² (36) = 1727.03, p < 0.001). Parallel analysis, scree plot inspection, and theoretical interpretability supported a three-factor solution (Appendix D). One item was removed due to cross-loading. The final model (Table 3) accounted for 68% of the variance (Factor 1: 26%, Factor 2: 24%, Factor 3: 17%) and showed good fit (CFI = 0.978, TLI = 0.911, RMSEA = 0.111, 90% CI (0.079, 0.146)).

**Table 3:** Factor Loadings of Environmental Items Env_Factor1: auditory characteristics of the preschool setting, particularly the prevalence of loud speech from teachers and peers; Env_Factor2: auditory characteristics of the home environment, including parental vocal expressions and disciplinary practices; Env_Factor3: the presence of family-related auditory experiences, such as exposure to parental conflicts and arguments at home

Item	Env_Factor1	Env_Factor2	Env_Factor3	Communality	Uniqueness	Complexity
I sometimes felt that kindergarten teachers spoke loudly	0.81	0.10	-0.06	0.67	0.33	1.00
I sometimes felt that my kindergarten friends spoke loudly	0.98	-0.04	0.00	0.93	0.07	1.00
I felt nervous when I heard friends arguing at kindergarten	0.54	-0.04	0.30	0.50	0.50	1.60
I thought my parents spoke loudly in daily life	0.25	0.43	0.19	0.45	0.55	2.00
My parents were strict	0.01	0.84	-0.03	0.69	0.31	1.00
I was often reprimanded loudly by my parents	-0.02	0.95	0.02	0.90	0.10	1.00
I felt nervous when I heard arguments at home	-0.01	0.01	1.00	1.01	-0.01	1.00
There were times when I stayed quiet to avoid disturbing my family's sleep	0.10	0.24	0.34	0.29	0.71	2.00

The three extracted factors represent distinct aspects of the early childhood auditory environment, reflecting exposure to different types of sound environments rather than individual sensitivity to sounds:

Factor 1 (Env_Factor1) represents auditory characteristics of the preschool setting, particularly the prevalence of loud speech from teachers and peers.

Factor 2 (Env_Factor2) reflects auditory characteristics of the home environment, including parental vocal expressions and disciplinary practices.

Factor 3 (Env_Factor3) captures the presence of family-related auditory experiences, such as exposure to parental conflicts and arguments at home.

The extracted factor scores (Env_Factor1, Env_Factor2, Env_Factor3) were computed for each participant and merged with the main dataset for subsequent regression analyses, where they were examined as predictors of auditory sensitivity outcomes.

Regression analyses

All VIF values were below 5, indicating no significant multicollinearity among predictors. Detailed VIF results are presented in Appendices E and F.

Core regression model

WLS1 (Sensitivity to Persistent Background Sounds)

Stepwise regression retained only Env_Factor2 as a significant predictor (β = 0.19, 95% CI (0.07, 0.31), p = 0.002), indicating that sensitivity to parental vocal expressions and disciplinary practices is positively associated with heightened responsiveness to subtle background sounds (Table 4).

**Table 4:** Regression Results for Auditory Sensitivity (WLS1 and WLS2) * Estimates and 95% confidence intervals (CIs) reported in the regression analyses refer to standardized beta coefficients. WLS1: sensitivity to subtle, non-salient sounds; WLS2: heightened reactivity to loud or sharp auditory stimuli; Env_Factor2: auditory characteristics of the home environment, including parental vocal expressions and disciplinary practices; Env_Factor3: the presence of family-related auditory experiences, such as exposure to parental conflicts and arguments at home

	WLS1		
Predictor	Estimate*	95% CI*	p-value
Env_Factor2	0.19	0.07-0.31	0.002
	WLS2		
Gender	0.50	0.27-0.72	<0.001
Env_Factor2	0.16	0.04-0.28	0.01
Env_Factor3	0.18	0.06-0.30	0.003

WLS2 (Responsiveness to Loud, Intrusive External Noises)

Stepwise regression identified gender (β = 0.50, 95% CI (0.27, 0.72), p < 0.001), Env_Factor2 (β = 0.16, 95% CI (0.04, 0.28), p = 0.01), and Env_Factor3 (β = 0.18, 95% CI (0.06, 0.30), p = 0.003) as significant predictors of WLS2. These results suggest that gender and sensitivity to both parental and conflict-related auditory cues contribute to elevated reactivity to loud environmental sounds.

Extended analysis with personality traits

WLS1 (Sensitivity to Persistent Background Sounds)

Stepwise regression identified four significant positive predictors: Being sensitive to stimuli (β = 0.23, 95% CI (0.09, 0.36), p = 0.001), wanting to stand out in all situations (β = 0.25, 95% CI (0.10, 0.40), p = 0.001), criticizing others when opinions differ (β = 0.24, 95% CI (0.10, 0.37), p < 0.001), and having trouble sleeping due to worries (β = 0.16, 95% CI (0.05, 0.27), p = 0.005). These results suggest that individuals with heightened reactivity, social assertiveness, and anxiety-related tendencies are more sensitive to background auditory input (Table 5).

**Table 5:** Regression Results of Personality Traits Predicting Auditory Sensitivity (WLS1 and WLS2) * Estimates and 95% confidence intervals (CIs) reported in the regression analyses refer to standardized beta coefficients. WLS1: sensitivity to subtle, non-salient sounds; WLS2: heightened reactivity to loud or sharp auditory stimuli

	WLS1		
Predictor	Estimate*	95% CI*	p-value
Being sensitive to stimuli	0.23	0.09 to 0.36	0.001
Wanting to stand out in all situations	0.25	0.10 to 0.40	0.001
Criticizing others when opinions differ	0.24	0.10 to 0.37	<0.001
Having trouble sleeping due to worries	0.16	0.05 to 0.27	0.005
	WLS2		
Being sensitive to stimuli	0.23	0.10 to 0.36	<0.001
Enjoying taking care of others	-0.18	-0.30 to -0.07	0.002
Feeling the urge to retaliate when insulted	0.14	0.04 to 0.24	0.008
Having trouble sleeping due to worries	0.15	0.04 to 0.27	0.006
Being prone to mental exhaustion	0.30	0.17 to 0.43	<0.001

WLS2 (Responsiveness to Loud, Intrusive External Noises)

Five personality traits emerged as significant predictors: Being sensitive to stimuli (β = 0.23, 95% CI (0.10, 0.36), p < 0.001), enjoying taking care of others (β = -0.18, 95% CI (-0.30, -0.07), p = 0.002), feeling the urge to retaliate when insulted (β = 0.14, 95% CI (0.04, 0.24), p = 0.008), having trouble sleeping due to worries (β = 0.15, 95% CI (0.04, 0.27), p = 0.006), and being prone to mental exhaustion (β = 0.30, 95% CI (0.17, 0.43), p < 0.001). These findings indicate that heightened sensory awareness, impulsive-reactive tendencies, and anxiety- or fatigue-related traits are all positively linked to increased responsiveness to intense auditory stimuli.

Non‑salient auditory stimuli

Chi-Square Analysis

More than 80% of participants did not perceive themselves as highly sensitive to the following everyday sounds: the sound of a toilet air dryer, indoor announcements, the sound of a refrigerator, the sound of flushing a toilet, the sound of water falling into a sink, the operation sound of a cash register, and the sound of a traffic signal (Appendix G).

Chi-square tests for all pairwise comparisons showed significant results (p < 0.01), indicating that these traits are interrelated rather than independent. This suggests that individuals exhibiting one of these traits are likely to show associations with others, reflecting shared personality tendencies related to sound sensitivity (Appendix H).

Item Analysis

All items retained for analysis had discrimination indices above 0.4 (Appendix I), indicating adequate item validity and suitability for subsequent factor extraction.

Factor Analysis

The selected items were subjected to factor analysis to derive a latent dimension of sensitivity to non-salient auditory stimuli. This factor was labeled as W1. Factor analysis further explored this structure. The KMO measure of sampling adequacy was 0.96, and Bartlett’s test of sphericity was highly significant (χ² (21) = 2609.39, p < 0.01), confirming that the correlation matrix was suitable for factor analysis. A single-factor solution was extracted, explaining 74% of the variance (Appendices A and J). All factor loadings exceeded 0.70, ranging from 0.73 to 0.93, indicating strong associations between these personality traits and the latent factor (Table 6).

**Table 6:** Factor Loading of Auditory Sensitivity Items (for Sounds That 80% of People Are Not Sensitive to) W1: sensitivity to ambient mechanical sounds that over 80% of participants rated as non-intrusive

Item	W1	Communality	Uniqueness	Complexity
Sound of a toilet air dryer	0.73	0.54	0.46	1.00
Indoor announcement	0.85	0.72	0.28	1.00
Sound of a refrigerator	0.89	0.79	0.21	1.00
Sound of flushing a toilet	0.89	0.80	0.20	1.00
Sound of water falling into a sink	0.84	0.70	0.30	1.00
Sound of a cash register operation	0.93	0.87	0.13	1.00
Sound of a traffic signal	0.88	0.78	0.22	1.00

Model fit indices supported the one-factor solution, with a root mean square residual (RMSR) of 0.02, a TLI of 0.96, and a CFI of 0.97. However, the RMSEA was 0.114 (90% CI: 0.091-0.139), suggesting a moderate fit. Given the strong factor loadings and the conceptual coherence of the extracted factor, it was labeled W1, representing a latent dimension of personality-related sensitivity to non-salient auditory stimuli. The computed factor scores for W1 were subsequently merged with the Q5 (personality traits) for further regression analyses, allowing for an examination of how this latent dimension of personality-related sensitivity to non-salient auditory stimuli interacts with other psychological characteristics.

Regression Analyses

Stepwise regression identified the following traits as significant predictors: Being sensitive to stimuli (β = 0.21, 95% CI (0.08-0.34), p = 0.002), maintaining a disciplined and orderly lifestyle (β = 0.16, 95% CI (0.03-0.25), p = 0.012), wanting to stand out in all situations (β = 0.29, 95% CI (0.15-0.44), p < 0.001), and criticizing others when opinions differ (β = 0.24, 95% CI (0.12-0.37), p < 0.001). Individuals with these traits were more likely to show heightened sensitivity to background sounds that are typically unnoticed by others (Table 7).

**Table 7:** Regression Analysis of Personality Traits Predicting Sensitivity to Sounds That Are Typically Not Perceived As Intrusive by Others * Estimates and 95% confidence intervals (CIs) reported in the regression analyses refer to standardized beta coefficients.

Predictor	Estimate*	95% CI*	p-value
Being sensitive to stimuli	0.21	0.08-0.34	0.002
Maintaining a disciplined and orderly lifestyle	0.16	0.03-0.25	0.012
Wanting to stand out in all situations	0.29	0.15-0.44	<0.001
Criticizing others when opinions differ	0.24	0.12-0.37	<0.001

In summary, the findings addressed both primary aims of the study: (1) two distinct dimensions of auditory sensitivity were successfully identified through factor analysis, and (2) these sensitivity profiles were found to be systematically associated with early childhood auditory experiences and stable personality traits, providing novel insight into individual variability in responses to everyday sounds.

## Discussion

The present study offers a comprehensive examination of auditory sensitivity by integrating environmental, musical, and personality determinants. Our findings suggest that auditory perception is a multidimensional construct, with individual differences shaped by both early-life experiences and enduring personality traits.

Environmental and developmental influences

Our analysis revealed that sensitivity to persistent background sounds (WLS1) is significantly predicted by sensitivity to parental vocal expressions and disciplinary practices (Env_Factor2). This finding suggests that early exposure to heightened auditory cues in the home environment, such as loud parental speech or reprimands, may foster an increased awareness of subtle ambient sounds. In contrast, sensitivity to loud, intrusive noises (WLS2) was most strongly associated with auditory cues related to familial conflict (Env_Factor3). This pattern implies that frequent exposure to conflict-related sounds may condition individuals to become hyper-responsive to external high-intensity auditory stimuli, possibly as an adaptive mechanism for detecting social threats. These observations align with prior research indicating that early auditory environments critically influence long-term sensory processing [35-37].

Personality as a modulator of auditory processing

Personality traits significantly shaped auditory sensitivity patterns. For background sounds (WLS1), being sensitive to stimuli, wanting to stand out, criticizing others, and having trouble sleeping due to worries were key predictors. These align with theories of sensory processing sensitivity and prior findings that link low agreeableness to intolerance for minor stressors and worry-related arousal to impaired noise habituation [38-40]. For loud, intrusive sounds (WLS2), sensitivity to stimuli again played a strong role [38]. Prosocial orientation (e.g., enjoying caregiving) was negatively associated, suggesting selective attention or emotional regulation benefits. Conversely, traits like retaliatory impulses, sleep-related worry, and mental exhaustion predicted greater reactivity-consistent with theories linking fatigue and cognitive load to reduced sensory filtering [41].

Sensitivity to non-salient auditory stimuli

A particularly novel aspect of this study is the identification of a latent factor representing sensitivity to non-salient auditory stimuli-sounds that most individuals typically ignore or filter out without distress. This factor was derived from items such as the sound of a refrigerator, the flushing of a toilet, water falling into a sink, and the operation of a cash register-ubiquitous environmental noises that are rarely perceived as disruptive. The unidimensional factor structure uncovered in our analysis suggests that sensitivity to these subtle sounds reflects a shared underlying trait of environmental hypersensitivity, possibly linked to heightened perceptual vigilance or difficulties in sensory habituation. The identification of this dimension highlights an often-overlooked form of auditory reactivity, extending current models beyond high-salience stimuli to capture finer-grained perceptual variability. Regression analyses further indicated that personality dimensions, especially those related to structured behavior and interpersonal assertiveness, are closely linked to this latent trait. This finding extends current models of sensory processing sensitivity by highlighting that even low-level, non-intrusive auditory stimuli are subject to individual perceptual variation based on personality [38].

Overall, these findings contribute to a clearer understanding of how everyday sound sensitivity can arise not solely from clinical conditions, but through complex interactions between personality traits and developmental experiences - an issue raised at the outset of this study.

Implications

The integrated framework provided by our study supports theories of embodied cognition and differential susceptibility, suggesting that perceptual experiences are dynamically shaped by both external environmental inputs and internal personality dispositions. These results have significant implications across several domains. Theoretically, our findings contribute to a deeper understanding of how early auditory experiences interact with personality traits to modulate sensory processing. Practically, they underscore the importance of creating adaptive environments in educational and occupational settings, for example, by implementing noise reduction strategies or designing spaces that accommodate individuals with heightened auditory sensitivity.

Furthermore, our study highlights the potential benefits of early interventions aimed at modifying adverse auditory environments. Such interventions could mitigate the development of auditory hypersensitivity later in life, particularly in individuals exposed to familial conflict or other stressors. Understanding these dynamics may also prove valuable in clinical contexts, such as for individuals with sensory processing disorders or autism spectrum disorder, where auditory hypersensitivity is a prevalent challenge [42].

Limitations

While our findings are robust, several limitations warrant consideration. First, the reliance on self-report measures may introduce bias, as auditory sensitivity is inherently subjective and shaped by individual interpretation. In particular, Q4 items assessing early childhood home environments rely on retrospective recall and may be susceptible to recall bias. However, it is unlikely that all of these associations can be explained simply by memory errors. For example, participants who remembered being frequently scolded by parents were more sensitive to background sounds, while those who recalled more family conflict were more reactive to loud and sudden noises. Second, the cross-sectional design limits causal inference. Longitudinal studies are needed to clarify how early auditory environments and personality traits interact over time to shape individual differences in auditory sensitivity. Additionally, although the questionnaire was developed through expert discussion and refined via pilot feedback, we did not formally assess content validity using quantitative indices such as the Content Validity Ratio (CVR), which may limit the psychometric rigor of the instrument.

Future research would benefit from more representative sampling methods, such as stratified or population-based sampling, to improve generalizability across age, region, and occupational background. Moreover, incorporating objective auditory assessments (e.g., psychoacoustic tasks or physiological markers) alongside self-report measures could strengthen the validity of findings. Further validation of the questionnaire using item response theory and confirmatory factor analysis is also recommended to ensure measurement precision.

Finally, while the sample offers insight into variability within non-clinical populations, it was obtained through voluntary online participation and may be subject to selection bias. Future research involving clinical groups, such as individuals with misophonia, autism spectrum disorder, or sensory processing disorders, could help validate and extend the current findings by examining whether the observed patterns of auditory sensitivity generalize to populations with known auditory processing challenges.

## Conclusions

In conclusion, our study advances the understanding of auditory perception by demonstrating that sensitivity to both background and intrusive sounds is a complex construct influenced by early environmental factors, musical experiences, and enduring personality traits. This multidimensional perspective enriches theoretical models of sensory processing and offers practical insights for tailoring environments to individual needs. Future research that bridges developmental neuroscience, personality psychology, and sensory processing will be essential in uncovering the full scope of factors that contribute to auditory sensitivity and its broader implications for well-being.

## Figures and Tables

**Figure 1:**
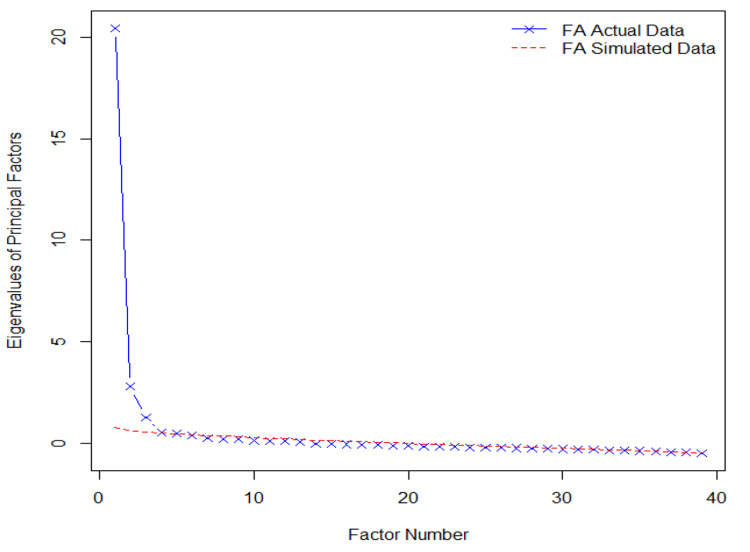
Parallel Analysis Scree Plot for the Auditory Environment

**Figure 2:**
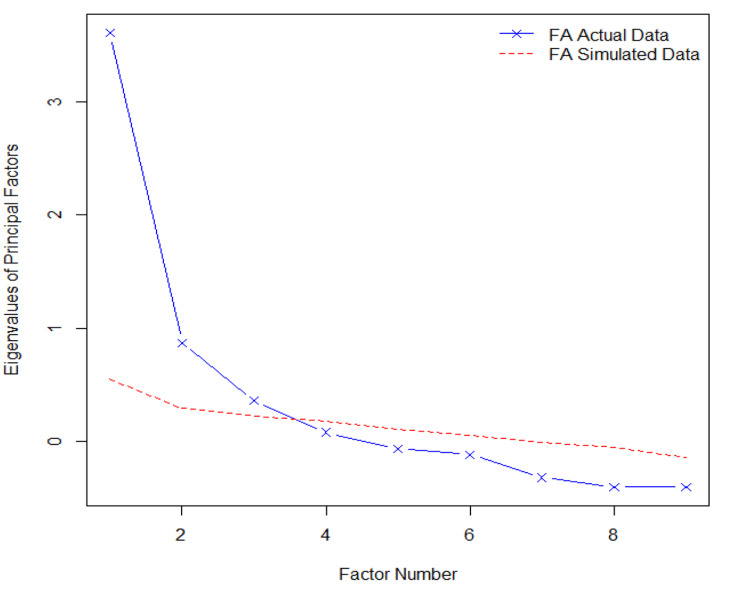
Parallel Analysis Scree Plot for the Preschool and Early Childhood Residential Environments

**Figure 3:**
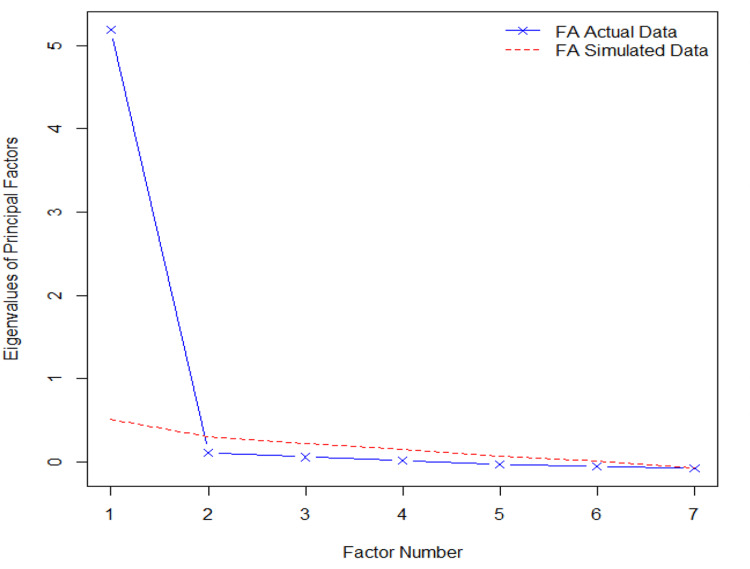
Parallel Analysis Scree Plot for the Auditory Environment (for Sounds That 80% of People Are Not Sensitive to)

## Appendices

Appendix A

**Table 8:** Item Analysis of the Auditory Environment

Row (Q6)	Missing	Mean	SD	Item Difficulty	Item Discrimination	α If Deleted
1. Sounds of large groups talking or crowd noise	0.00%	1.62	0.94	0.54	0.59	0.97
2. Sounds of campaign vehicles or advertising trucks	0.00%	1.73	0.97	0.58	0.57	0.97
3. Construction site noise	0.00%	1.81	0.99	0.6	0.57	0.97
4. Motorcycle or car horns	0.00%	1.97	0.98	0.66	0.58	0.97
5. Sounds of a baby crying	0.00%	1.56	1.07	0.52	0.50	0.97
6. Sounds of game centers or bowling alleys	0.00%	1.29	0.98	0.43	0.61	0.97
7. Vacuum cleaner noise	0.00%	1.27	0.98	0.42	0.66	0.97
8. Ambulance siren	0.00%	1.59	1.02	0.53	0.60	0.97
9. Sound of a running train	0.00%	1.28	0.99	0.43	0.64	0.97
10. Sound of a toilet air dryer	0.00%	0.83	0.85	0.28	0.65	0.97
11. Sound of a helicopter	0.00%	1.23	0.98	0.41	0.66	0.97
12. Sound of festival taiko drums	0.00%	1.25	1.05	0.42	0.64	0.97
13. Sound from TV or radio	0.00%	1.04	0.95	0.35	0.65	0.97
14. Sound of dishes clinking	0.00%	1.62	1.06	0.54	0.60	0.97
15. Sound of train brakes	0.00%	1.57	1.06	0.52	0.60	0.97
16. Sound indicating train departure or arrival	0.00%	1.23	0.98	0.41	0.70	0.96
17. Sound of a hairdryer	0.00%	1.01	0.95	0.34	0.68	0.97
18. Sound of a phone ringing	0.00%	1.46	1.05	0.49	0.68	0.97
19. Sound of a railroad crossing	0.00%	1.30	1.06	0.43	0.73	0.96
20. Background music in a store	0.00%	0.91	0.92	0.30	0.70	0.97
21. Indoor announcement	0.00%	0.84	0.87	0.28	0.75	0.96
22. Sound of audio equipment	0.00%	0.92	0.92	0.31	0.69	0.97
23. Sound of air conditioning or ventilation fan	0.00%	0.88	0.89	0.29	0.69	0.97
24. Sound of a door opening or closing	0.00%	1.10	1.00	0.37	0.68	0.97
25. Sound indicating a bus stopping or entering	0.00%	0.96	0.93	0.32	0.74	0.96
26. Sound of a clock's second hand	0.00%	0.94	0.98	0.31	0.62	0.97
27. Sound of a cough	0.00%	1.32	1.05	0.44	0.64	0.97
28. Sound of a refrigerator	0.00%	0.69	0.83	0.23	0.71	0.97
29. Footsteps inside a house	0.00%	1.02	1.01	0.34	0.63	0.97
30. Sound of flushing a toilet	0.00%	0.80	0.89	0.27	0.74	0.96
31. Sound of sniffling	0.00%	1.14	1.00	0.38	0.60	0.97
32. Sound of knocking on a door	0.00%	0.98	0.93	0.33	0.71	0.96
33. Sound of water falling into a sink	0.00%	0.84	0.90	0.28	0.68	0.97
34. Sound of a cash register operation	0.00%	0.69	0.82	0.23	0.70	0.97
35. Sound of a traffic signal	0.00%	0.73	0.85	0.24	0.69	0.97
36. Whispering voices between others	0.00%	1.44	1.08	0.48	0.61	0.97
37. Casual conversation in a public place	0.00%	1.52	1.08	0.51	0.61	0.97
38. Sound of writing utensils during a test	0.00%	1.18	1.03	0.39	0.60	0.97
39. Shouting voice	0.00%	2.13	1.01	0.71	0.48	0.97
Mean inter-item correlation = 0.432; Cronbach's α = 0.967						

Appendix B

Appendix C 

**Table 9:** Item Analysis of the Preschool and Early Childhood Residential Environments

Row (Q3, Q4)	Missing	Mean	SD	Item Difficulty	Item Discrimination	α If Deleted
Q3						
1. The kindergarten environment in early childhood was active in sports	0.00%	2.21	0.97	0.55	0.22	0.79
2. The kindergarten was located in an urban area	0.00%	0.96	1.14	0.24	0.24	0.79
3. I was often scolded by kindergarten teachers	0.00%	0.94	1.12	0.24	0.35	0.79
4. Birds and insects could be heard indoors at the kindergarten	0.00%	1.79	1.28	0.45	0.28	0.79
5. Noises from cars, trains, and helicopters could be heard indoors at the kindergarten	0.00%	1.32	1.30	0.33	0.40	0.78
6. I sometimes felt that kindergarten teachers spoke loudly	0.00%	1.1	1.28	0.28	0.51	0.78
7. I sometimes felt that my kindergarten friends spoke loudly	0.00%	1.3	1.33	0.32	0.50	0.78
8. I felt nervous when I heard friends arguing at kindergarten	0.00%	1.47	1.39	0.37	0.45	0.78
Q4						
1. The childhood living environment was quiet	0.00%	1.98	0.95	0.49	-0.01	0.80
2. I was raised in an urban area	0.26%	1.04	1.15	0.26	0.20	0.80
3. There was frequent interaction with neighbors	0.79%	1.81	1.02	0.45	0.09	0.80
4. Birds and insects could be heard indoors at home	0.00%	1.94	1.10	0.48	0.29	0.79
5. Noises from cars, trains, and helicopters could be heard indoors at home	0.53%	1.65	1.12	0.41	0.33	0.79
6. I often played outside	0.26%	2.33	0.90	0.58	0.17	0.80
7. I thought my parents spoke loudly in daily life	0.79%	1.19	1.14	0.30	0.47	0.78
8. My parents were strict	0.26%	1.44	1.09	0.36	0.53	0.78
9. I was often reprimanded loudly by my parents	0.26%	1.39	1.11	0.35	0.52	0.78
10. The household was lively	0.00%	1.91	0.97	0.48	0.25	0.79
11. My parents frequently argued	1.05%	1.19	1.30	0.30	0.39	0.78
12. Fights with my siblings were intense (only if applicable)	14.47%	1.61	1.16	0.40	0.29	0.79
13. I felt nervous when I heard arguments at home	0.00%	1.53	1.29	0.38	0.46	0.78
14. There were times when I stayed quiet to avoid disturbing my family's sleep	0.00%	1.36	1.27	0.34	0.39	0.78
15. I think my parents liked music	0.53%	2.06	0.97	0.51	0.25	0.79
Mean inter-item correlation = 0.138; Cronbach's α = 0.795						

Appendix D

Appendix E

**Table 10:** Variance Inflation Factors (VIFs) for Predictors Included in Regression Models of Auditory Sensitivity Env_Factor1: auditory characteristics of the preschool setting, particularly the prevalence of loud speech from teachers and peers; Env_Factor2: auditory characteristics of the home environment, including parental vocal expressions and disciplinary practices; Env_Factor3: the presence of family-related auditory experiences, such as exposure to parental conflicts and arguments at home

Variable	VIF
Age	1.05
Gender	1.16
Env_Factor1	1.34
Env_Factor2	1.24
Env_Factor3	1.43
Music Experience Score	1.11

Appendix F

**Table 11:** Variance Inflation Factors (VIFs) for Q5 Personality Trait Items in Multiple Regression Model

Variable	VIF
1. Taking the perspective of others	1.49
2. Being sensitive to stimuli	1.50
3. Feeling compelled to help those in distress	1.47
4. Understanding others' suffering	1.53
5. Finding it difficult to refuse requests	1.48
6. Disliking self-sacrifice for others	1.73
7. Enjoying taking care of others	1.70
8. Preferring to focus on oneself rather than others	1.37
9. Enjoying devoting oneself to others	1.86
10. Feeling sympathy for those in unfortunate situations	1.45
11. Planning trips in detail	1.78
12. Keeping desks and workplaces organized	1.47
13. Following a structured approach to tasks	1.98
14. Maintaining a disciplined and orderly lifestyle	1.60
15. Writing well-structured texts	1.46
16. Writing neatly and carefully	1.49
17. Organizing and keeping letters in order	1.71
18. Always making a plan before taking action	2.01
19. Keeping books in designated places on bookshelves	1.55
20. Eating meals at fixed times	1.44
21. Desiring to be the center of attention	2.34
22. Enjoying sharing personal experiences in public	1.81
23. Making an effort to dress differently from others	1.27
24. Wanting to take the lead role in a play	2.27
25. Wanting to stand out in all situations	1.63
26. Aspiring to win competitions	1.49
27. Wanting to associate with famous people	1.47
28. Feeling dissatisfied when not recognized by others	1.56
29. Enjoying being talked about by others	1.81
30. Liking to be flattered and praised	1.76
31. Having strong personal preferences	1.44
32. Always responding when criticized	1.62
33. Being tolerant towards others	1.45
34. Feeling the urge to retaliate when insulted	1.57
35. Becoming easily excited	1.75
36. Criticizing others when opinions differ	1.68
37. Not staying silent when treated rudely	1.61
38. Having a short temper	1.90
39. Frequently taking frustrations out on others	1.78
40. Blaming others when things go wrong	1.93
41. Being prone to excessive worry	2.36
42. Being overly concerned with small matters	2.87
43. Tending to overcomplicate things	2.32
44. Being highly neurotic	2.33
45. Overanalyzing others' words and actions	1.87
46. Not being particularly fixated on things	1.41
47. Having trouble sleeping due to worries	1.77
48. Easily forgetting unpleasant experiences	1.65
49. Being prone to mental exhaustion	1.94
50. Dwelling on failures for a long time	2.21

Appendix G

**Table 12:** Response Proportions for the Auditory Environment

Variable	Not Sensitive (%)	Not Very Sensitive (%)	Somewhat Sensitive (%)	Extremely Sensitive (%)
1. Sounds of large groups talking or crowd noise	14	29	39	18
2. Sounds of campaign vehicles or advertising trucks	12	27	35	25
3. Construction site noise	12	25	34	29
4. Motorcycle or car horns	10	19	34	37
5. Sounds of a baby crying	21	28	27	24
6. Sounds of game centers or bowling alleys	23	37	26	14
7. Vacuum cleaner noise	25	38	24	14
8. Ambulance siren	17	28	33	22
9. Sound of a running train	25	35	26	14
10. Sound of a toilet air dryer	41	40	14	5
11. Sound of a helicopter	27	35	26	12
12. Sound of festival taiko drums	31	28	26	15
13. Sound from TV or radio	34	36	20	9
14. Sound of dishes clinking	19	24	32	24
15. Sound of train brakes	20	27	29	24
16. Sound indicating train departure or arrival	26	38	23	13
17. Sound of a hairdryer	36	37	19	9
18. Sound of a phone ringing	23	28	29	20
19. Sound of a railroad crossing	27	33	21	18
20. Background music in a store	40	36	17	7
21. Indoor announcement	41	39	15	5
22. Sound of audio equipment	39	38	16	8
23. Sound of air conditioning or ventilation fan	40	39	14	7
24. Sound of a door opening or closing	34	34	20	12
25. Sound indicating a bus stopping or entering	37	39	15	9
26. Sound of a clock's second hand	41	32	17	9
27. Sound of a cough	28	28	28	16
28. Sound of a refrigerator	50	35	10	4
29. Footsteps inside a house	38	33	17	12
30. Sound of flushing a toilet	45	36	13	6
31. Sound of sniffling	31	36	20	13
32. Sound of knocking on a door	37	37	19	7
33. Sound of water falling into a sink	42	39	12	7
34. Sound of a cash register operation	49	39	7	6
35. Sound of a traffic signal	48	36	11	5
36. Whispering voices between others	25	27	27	21
37. Casual conversation in a public place	23	26	28	23
38. Sound of writing utensils during a test	31	33	21	14
39. Shouting voice	10	16	25	49

Appendix H 

**Table 13:** Chi-Square Analysis (for Sounds That 80% of People Are Not Sensitive to)

Variable 1	Variable 2	Chi-Square	p-value
Sound of a toilet air dryer	Indoor announcement	206.69	<0.01
Sound of a toilet air dryer	Sound of a refrigerator	233.18	<0.01
Sound of a toilet air dryer	Sound of flushing a toilet	255.65	<0.01
Sound of a toilet air dryer	Sound of water falling into a sink	208.05	<0.01
Sound of a toilet air dryer	Sound of a cash register operation	258.11	<0.01
Sound of a toilet air dryer	Sound of a traffic signal	199.41	<0.01
Indoor announcement	Sound of a refrigerator	349.27	<0.01
Indoor announcement	Sound of flushing a toilet	345.15	<0.01
Indoor announcement	Sound of water falling into a sink	229.86	<0.01
Indoor announcement	Sound of a cash register operation	329.78	<0.01
Indoor announcement	Sound of a traffic signal	340.31	<0.01
Sound of a refrigerator	Sound of flushing a toilet	407.86	<0.01
Sound of a refrigerator	Sound of water falling into a sink	351.68	<0.01
Sound of a refrigerator	Sound of a cash register operation	418.91	<0.01
Sound of a refrigerator	Sound of a traffic signal	396.68	<0.01
Sound of flushing a toilet	Sound of water falling into a sink	316.14	<0.01
Sound of flushing a toilet	Sound of a cash register operation	360.85	<0.01
Sound of flushing a toilet	Sound of a traffic signal	350.98	<0.01
Sound of water falling into a sink	Sound of a cash register operation	399.25	<0.01
Sound of water falling into a sink	Sound of a traffic signal	338.11	<0.01
Sound of a cash register operation	Sound of a traffic signal	472.10	<0.01

Appendix I 

**Table 14:** Item Analysis of the Auditory Environment (for Sounds That 80% of People Are Not Sensitive to)

Row (Q6)	Missing	Mean	SD	Item Difficulty	Item Discrimination	α If Deleted
10. Sound of a toilet air dryer	0.00%	0.83	0.85	0.28	0.63	0.93
21. Indoor announcement	0.00%	0.84	0.87	0.28	0.76	0.91
28. Sound of a refrigerator	0.00%	0.69	0.83	0.23	0.80	0.91
30. Sound of flushing a toilet	0.00%	0.80	0.89	0.27	0.80	0.91
33. Sound of water falling into a sink	0.00%	0.84	0.9	0.28	0.74	0.92
34. Sound of a cash register operation	0.00%	0.69	0.82	0.23	0.84	0.91
35. Sound of a traffic signal	0.00%	0.73	0.85	0.24	0.78	0.91
Mean inter-item correlation = 0.639; Cronbach's α = 0.925						

Appendix J 

Appendix K 

**Table 15:** Questionnaire Items

Item
Age
Gender
Q2: Early Childhood Musical Experiences
1. Listening to music during early childhood
2. Watching children's television programs during early childhood
3. Experiencing eurhythmics (moving the body to music) during early childhood
4. Singing songs in preschool
5. Playing the keyboard harmonica in preschool
6. Practicing instrumental ensemble performances, such as in a preschool marching band
7. At what age did you begin learning a musical instrument other than the piano or electronic organ?
Q3: Preschool Environment
1. The kindergarten environment in early childhood was active in sports
2. The kindergarten was located in an urban area
3. I was often scolded by kindergarten teachers
4. Birds and insects could be heard indoors at the kindergarten
5. Noises from cars, trains, and helicopters could be heard indoors at the kindergarten
6. I sometimes felt that kindergarten teachers spoke loudly
7. I sometimes felt that my kindergarten friends spoke loudly
8. I felt nervous when I heard friends arguing at kindergarten
Q4: Early Childhood Residential Environment
1. The childhood living environment was quiet
2. I was raised in an urban area
3. There was frequent interaction with neighbors
4. Birds and insects could be heard indoors at home
5. Noises from cars, trains, and helicopters could be heard indoors at home
6. I often played outside
7. I thought my parents spoke loudly in daily life
8. My parents were strict
9. I was often reprimanded loudly by my parents
10. The household was lively
11. My parents frequently argued
12. Fights with my siblings were intense (only if applicable)
13. I felt nervous when I heard arguments at home
14. There were times when I stayed quiet to avoid disturbing my family's sleep
15. I think my parents liked music
Q5: Personality Traits
1. Taking the perspective of others
2. Being sensitive to stimuli
3. Feeling compelled to help those in distress
4. Understanding others' suffering
5. Finding it difficult to refuse requests
6. Disliking self-sacrifice for others
7. Enjoying taking care of others
8. Preferring to focus on oneself rather than others
9. Enjoying devoting oneself to others
10. Feeling sympathy for those in unfortunate situations
11. Planning trips in detail
12. Keeping desks and workplaces organized
13. Following a structured approach to tasks
14. Maintaining a disciplined and orderly lifestyle
15. Writing well-structured texts
16. Writing neatly and carefully
17. Organizing and keeping letters in order
18. Always making a plan before taking action
19. Keeping books in designated places on bookshelves
20. Eating meals at fixed times
21. Desiring to be the center of attention
22. Enjoying sharing personal experiences in public
23. Making an effort to dress differently from others
24. Wanting to take the lead role in a play
25. Wanting to stand out in all situations
26. Aspiring to win competitions
27. Wanting to associate with famous people
28. Feeling dissatisfied when not recognized by others
29. Enjoying being talked about by others
30. Liking to be flattered and praised
31. Having strong personal preferences
32. Always responding when criticized
33. Being tolerant towards others
34. Feeling the urge to retaliate when insulted
35. Becoming easily excited
36. Criticizing others when opinions differ
37. Not staying silent when treated rudely
38. Having a short temper
39. Frequently taking frustrations out on others
40. Blaming others when things go wrong
41. Being prone to excessive worry
42. Being overly concerned with small matters
43. Tending to overcomplicate things
44. Being highly neurotic
45. Overanalyzing others' words and actions
46. Not being particularly fixated on things
47. Having trouble sleeping due to worries
48. Easily forgetting unpleasant experiences
49. Being prone to mental exhaustion
50. Dwelling on failures for a long time
Q6: Auditory Environment and Sensitivity
1. Sounds of large groups talking or crowd noise
2. Sounds of campaign vehicles or advertising trucks
3. Construction site noise
4. Motorcycle or car horns
5. Sounds of a baby crying
6. Sounds of game centers or bowling alleys
7. Vacuum cleaner noise
8. Ambulance siren
9. Sound of a running train
10. Sound of a toilet air dryer
11. Sound of a helicopter
12. Sound of festival taiko drums
13. Sound from TV or radio
14. Sound of dishes clinking
15. Sound of train brakes
16. Sound indicating train departure or arrival
17. Sound of a hairdryer
18. Sound of a phone ringing
19. Sound of a railroad crossing
20. Background music in a store
21. Indoor announcement
22. Sound of audio equipment
23. Sound of air conditioning or ventilation fan
24. Sound of a door opening or closing
25. Sound indicating a bus stopping or entering
26. Sound of a clock's second hand
27. Sound of a cough
28. Sound of a refrigerator
29. Footsteps inside a house
30. Sound of flushing a toilet
31. Sound of sniffling
32. Sound of knocking on a door
33. Sound of water falling into a sink
34. Sound of a cash register operation
35. Sound of a traffic signal
36. Whispering voices between others
37. Casual conversation in a public place
38. Sound of writing utensils during a test
39. Shouting voice
